# Supplementary material for: Strategic human resource management practitioners’ emotional intelligence and affective organizational commitment in higher education institutions in Georgia during post-COVID-19
Source: PLoS One. 2023 Dec 22;18(12):e0295084. doi: 10.1371/journal.pone.0295084 (PMC10745210; doi:10.1371/journal.pone.0295084)
Supplement: S1 Appendix — (DOCX) [file pone.0295084.s001.docx]

**Appendix 1:**

**Methods and sampling**

The area of this study is the capital of Georgia which includes the largest number of public (12) and private universities (30). The population of this study includes all managers of universities responsible for decision making in HR area in all public and private universities both situated in Tbilisi, Georgia. Purposive sampling technique based on availability will be adopted by the researchers in selecting the accessible human resource managers across the universities in the zone. Thus, a total of 393 HR directors, HR managers and HR officers will be selected based on their availability during data collection. The breakdown of the sample is presented in the following table. All managers will be included in the study, and approximately sample is 393.

| **Public Universities** | **Location** | **Sample** |
| --- | --- | --- |
| [Akhaltsikhe State Education University](https://en.wikipedia.org/w/index.php?title=Akhaltsikhe_State_Education_University&action=edit&redlink=1) | Tbilisi | 15 |
| [Georgian Technical University](https://en.wikipedia.org/wiki/Georgian_Technical_University) | Tbilisi | 17 |
| [Gori State Teaching University](https://en.wikipedia.org/wiki/Gori_State_Teaching_University) | Tbilisi | 14 |
| [Ilia State University](https://en.wikipedia.org/wiki/Ilia_State_University) | Tbilisi | 20 |
| [International School of Economics at Tbilisi State University](https://en.wikipedia.org/wiki/International_School_of_Economics_at_Tbilisi_State_University) | Tbilisi | 17 |
| [Sokhumi State University](https://en.wikipedia.org/wiki/Sokhumi_State_University) | Tbilisi | 10 |
| [Tbilisi Ivane Javakhishvili State University](https://en.wikipedia.org/wiki/Tbilisi_Ivane_Javakhishvili_State_University) | Tbilisi | 14 |
| [Tbilisi State Academy of Arts](https://en.wikipedia.org/wiki/Tbilisi_State_Academy_of_Arts) | Tbilisi | 5 |
| [Tbilisi State Medical University](https://en.wikipedia.org/wiki/Tbilisi_State_Medical_University) | Tbilisi | 12 |
| [Tbilisi Vano Sarajishvili State Conservatory](https://en.wikipedia.org/wiki/Tbilisi_Vano_Sarajishvili_State_Conservatory) | Tbilisi | 6 |
| [Telavi Iakob Gogebashvili State University](https://en.wikipedia.org/wiki/Telavi_Iakob_Gogebashvili_State_University) | Tbilisi | 8 |
| [Shota Rustaveli University of Theater and Cinema](https://en.wikipedia.org/wiki/Theatre_and_Film_University_(Georgia)) | Tbilisi | 7 |
| **Private Universities** | **Tbilisi** | **Sample** |
| [Agricultural University of Georgia](https://en.wikipedia.org/wiki/Agricultural_University_of_Georgia) (Tbilisi) | Tbilisi | 5 |
| [American University for Humanities Tbilisi Campus](https://en.wikipedia.org/w/index.php?title=American_University_for_Humanities_Tbilisi_Campus&action=edit&redlink=1) | Tbilisi | 10 |
| [Business and Technology University](https://en.wikipedia.org/w/index.php?title=Business_and_Technology_University&action=edit&redlink=1) | Tbilisi | 12 |
| [Caucasus International University](https://en.wikipedia.org/w/index.php?title=Caucasus_International_University&action=edit&redlink=1) | Tbilisi | 15 |
| [Caucasus University](https://en.wikipedia.org/wiki/Caucasus_University) | Tbilisi | 8 |
| [David Aghmashenebeli University of Georgia](https://en.wikipedia.org/wiki/David_Aghmashenebeli_University_of_Georgia) | Tbilisi | 5 |
| [David Tvildiani Medical University](https://en.wikipedia.org/w/index.php?title=David_Tvildiani_Medical_University&action=edit&redlink=1) | Tbilisi | 7 |
| [East European University](https://en.wikipedia.org/w/index.php?title=East_European_University&action=edit&redlink=1) | Tbilisi | 7 |
| [European University](https://en.wikipedia.org/w/index.php?title=European_University_(Tbilisi)&action=edit&redlink=1) (formerly [European Teaching University](https://en.wikipedia.org/w/index.php?title=European_Teaching_University&action=edit&redlink=1) Tbilisi) | Tbilisi | 12 |
| [Free University of Tbilisi](https://en.wikipedia.org/wiki/Free_University_of_Tbilisi) | Tbilisi | 10 |
| [Georgian American University](https://en.wikipedia.org/w/index.php?title=Georgian_American_University&action=edit&redlink=1) | Tbilisi | 8 |
| [Georgian Aviation University](https://en.wikipedia.org/wiki/Georgian_Aviation_University) | Tbilisi | 5 |
| [Georgian Institute of Public Affairs](https://en.wikipedia.org/wiki/Georgian_Institute_of_Public_Affairs) | Tbilisi | 6 |
| [Grigol Robakidze University](https://en.wikipedia.org/wiki/Grigol_Robakidze_University) | Tbilisi | 5 |
| [Guram Tavartkiladze Teaching University](https://en.wikipedia.org/w/index.php?title=Guram_Tavartkiladze_Teaching_University&action=edit&redlink=1) | Tbilisi | 6 |
| [New Vision University](https://en.wikipedia.org/w/index.php?title=New_Vision_University&action=edit&redlink=1) | Tbilisi | 8 |
| [Petre Shotadze Tbilisi Medical Academy](https://en.wikipedia.org/wiki/Tbilisi_Medical_Academy) | Tbilisi | 12 |
| [Saint Andrews Georgian University](https://en.wikipedia.org/w/index.php?title=Saint_Andrews_Georgian_University&action=edit&redlink=1) | Tbilisi | 5 |
| [Tbilisi Medical Academy](https://en.wikipedia.org/wiki/Tbilisi_Medical_Academy) | Tbilisi | 6 |
| [Tbilisi Medical Institute “Hippocrates”](https://en.wikipedia.org/w/index.php?title=Tbilisi_Medical_Institute_%22Hippocrates%22&action=edit&redlink=1) | Tbilisi | 7 |
| [Tbilisi Teaching University](https://en.wikipedia.org/w/index.php?title=Tbilisi_Teaching_University&action=edit&redlink=1) | Tbilisi | 8 |
| [Tbilisi University “Metekhi”](https://en.wikipedia.org/w/index.php?title=Tbilisi_University_%22Metekhi%22&action=edit&redlink=1) | Tbilisi | 7 |
| [Teaching University European Academy](https://en.wikipedia.org/w/index.php?title=Teaching_University_European_Academy&action=edit&redlink=1) | Tbilisi | 6 |
| [Teaching University Geomedi](http://www.geomedi.edu.ge/) | Tbilisi | 10 |
| [Teaching University “Iveria”](https://en.wikipedia.org/w/index.php?title=Teaching_University_%22Iveria%22&action=edit&redlink=1) | Tbilisi | 7 |
| [Teaching University of International Relations of Georgia](https://en.wikipedia.org/w/index.php?title=Teaching_University_of_International_Relations_of_Georgia&action=edit&redlink=1) (Tbilisi) | Tbilisi | 9 |
| [Teaching University SEU](https://en.wikipedia.org/w/index.php?title=Teaching_University_SEU&action=edit&redlink=1) | Tbilisi | 12 |
| [The University of Georgia](https://en.wikipedia.org/wiki/University_of_Georgia_(Tbilisi)) | Tbilisi | 15 |
| [University “Sakartvelo”](https://en.wikipedia.org/w/index.php?title=University_%22Sakartvelo%22&action=edit&redlink=1) | Tbilisi | 10 |
| [Alterbridge University](https://alterbridge.edu.ge/) | Tbilisi | 5 |
|  | Total | 393 |
