# Supplementary material for: Strategic human resource management practitioners’ emotional intelligence and affective organizational commitment in higher education institutions in Georgia during post-COVID-19
Source: PLoS One. 2023 Dec 22;18(12):e0295084. doi: 10.1371/journal.pone.0295084 (PMC10745210; doi:10.1371/journal.pone.0295084)
Supplement: S2 Appendix — (DOCX) [file pone.0295084.s002.docx]

**Appendix 2: Variables and measures**

| **SHRM**  **17 questions**  Kutieshat and Farmanesh (2022) &  Bieńkowska et al (2022).  Kutieshat R, Farmanesh P. The Impact of New Human Resource Management Practices on Innovation Performance during the COVID 19 Crisis: A New Perception on Enhancing the Educational Sector. Sustainability. 2022; 14(5):2872. <https://doi.org/10.3390/su14052872>.  Bieńkowska A, Koszela A, Sałamacha A, Tworek K. COVID-19 oriented HRM strategies influence on job and organizational performance through job-related attitudes. PLoS One. 2022 Apr 13;17(4):e0266364. doi: 10.1371/journal.pone.0266364. PMID: 35417468; PMCID: PMC9007351. | **Covid -19**  **Staffing**  Kutieshat and Farmanesh (2022) | Necessary actions are being taken by the HR department to avoid layoffs in post COVID-19 environment. |
| --- | --- | --- |
|  |  | The HR department hiring procedure is more efficient due to the adoption of E-recruitment. in post COVID-19 environment |
|  |  | Adoption of E-HRM portal to maintain the employee’s record and information in post COVID-19 environment. |
|  |  | HR department is reorganizing employees to appropriate positions effectively as per situations in post COVID-19 environment |
|  | **Covid -19**  **Training**  Kutieshat and Farmanesh (2022) |  |
|  |  | Appropriate job training set for employees by the organization in post COVID-19 environment |
|  |  | Training of new skills and technology to compete in the learning industry [is provided] in post COVID-19 environment. |
|  | **Covid -19**  **Performance appraisal**  Bieńkowska et al (2022). | The HR department keeps employees informed about the work issues as well as its performance in post COVID-19 environment |
|  |  | Employees always complete the duties specified in their job description in post COVID-19 environment |
|  |  | Employees always meet all the formal performance requirements of their job in post COVID-19 environment |
|  |  | Employees complete their tasks efficiently in post COVID-19 environment |
|  |  | Employees are always able to overcome obstacles to complete their tasks in post COVID-19 environment |
|  |  | Employees are rarely absent from my work in post COVID-19 environment |
|  |  | Employees avoid absenteeism at work without a legitimate reason in post COVID-19 environment |
|  |  | Employees make few mistakes at work in post COVID-19 environment |
|  | **Covid -19**  **Compensation**  Kutieshat and Farmanesh (2022) | The effort which I put in my job that fairly rewarded in post COVID-19 Environment |
|  |  | One’s contribution recognized reflects the fairness of reward system in post COVID-19 environment |
|  |  | Individual performance-based reward system in post COVID-19 environment |
| **OC 6**  Allen and Meyer (1990).  Allen, N.J. and Meyer, J.P. (1990) The Measurement and Antecedents of Affective, Continuance, and Normative Commitment to the Organization. Journal of Occupational Psychology, 63, 1-18 | **Affective Organizational Commitment** | Employees feel a strong sense of belonging to their organization |
|  |  | Employees feel personally attached to their work organization |
|  |  | Employees proud to tell others to work at their organization |
|  |  | Working at our organization has a great deal of personal meaning to employees |
|  |  | Employees would be happy to work at their organization until retire |
|  |  | Employee really feel that problems faced by organization are also their problems |
| **PC 17**  Millward and Hopkins (1998)  Millward, L. J., & Hopkins, L. J. (1998). Psychological Contracts, Organizational and Job Commitment. Journal of Applied Social Psychology, 28, 1530-1556. | **Transactional** | Employees come to work purely to get the job done |
|  |  | Employees do their job just for the money |
|  |  | Employees only do what is necessary to get the job done |
|  |  | Employee work to achieve the purely short-term goals of their job |
|  |  | Loyalty of employees to the organisation is defined by the terms of their contract |
|  |  | Employees is motivated to contribute 100% to this company in return for future employment benefits |
|  |  | Employees prefer to work a strictly defined set of working hours |
|  |  | It is important not to get too involved in their job |
|  |  | Employees expect to be paid for any overtime their do |
|  |  | Employees career path in the organisation is clearly mapped out |
|  | **Rational** | Employees expect to grow in their organisation |
|  |  | Employees expect to gain promotion in this company with length of service and effort to achieve goals |
|  |  | Employees feel this company reciprocates the effort put in by organization |
|  |  | Employees heavily involved in their place of work |
|  |  | Employees feel part of a team in this organisation |
|  |  | Employees have a reasonable chance of promotion if their work hard |
|  |  | Employees will work for their company indefinitely |
| **EI 16**  Law, K. S., Wong, C. S., & Song, L. J. (2004). The construct and criterion validity of emotional intelligence and its potential utility for management studies, Journal of applied psychology, 89(3),483-496. | **Self-awareness** | I know how to label my emotions |
|  |  | I am aware of the thoughts that influence my emotions |
|  |  | I differentiate one emotion from another |
|  |  | I know how my emotions influence what I do |
|  | **Self management and motivation** | I know how to motivate myself |
|  |  | I have my goals clear |
|  |  | I pursue my objectives despite the difficulties |
|  | **Decision making** | I make decisions analyzing carefully possible consequences |
|  |  | I usually consider advantages and disadvantages of each option before I make decisions |
|  |  | I do not make decisions carelessly |
|  | **Self-awareness and prosocial behaviour** | I know what people expect from others |
|  |  | I pay attention to the needs of others |
|  |  | I usually know how to help others who need that |
|  |  | I have good relationships with my workmates |
|  |  | I usually listen in an active way |
|  |  | I offer help to those who need me |
