# Supplementary material for: Strategic human resource management practitioners’ emotional intelligence and affective organizational commitment in higher education institutions in Georgia during post-COVID-19
Source: PLoS One. 2023 Dec 22;18(12):e0295084. doi: 10.1371/journal.pone.0295084 (PMC10745210; doi:10.1371/journal.pone.0295084)
Supplement: Attachment — Submitted filename: Response to Reviewers.docx [file pone.0295084.s003.docx]

Dear Editor,

The manuscript has been revised according to the suggestions and comments of the reviewers.

Please kindly note that a new analysis has been added and the major revised parts are highlighted in yellow color for your convenience of re-reviewing. I hope that it's now suitable for publication.

| REVIEWER 1 COMMENT | AUTHOR RESPONSE | PAGE NUMBER |
| --- | --- | --- |
| Although, author has found positive correlations between EI and SHRM (hypothesis 1) and SHRM and PC (hypothesis 3) mentioned at line number 561 and 563, but these correlations are not very strong. Therefore reasons for the same may be explained. | Thank you very much. We appreciate the reviewer’s insightful suggestion. In cases of H1 and H3 were identified not strong [or weak] but statistically significant correlations. In essence, finding a weak correlation that is statically significant suggests that particular exposure has an impact on the outcome variable, but that there are other important determinants as well (LaMorte, 2021). Among those factors: supervisor support, workload, leadership styles which were not included in this study could influence PC, EI and SHRM.  LaMorte, W. evaluation association between two continuance variables. Boston University School of Public Health, 2021. https://sphweb.bumc.bu.edu | 24 |

We are thankful to the reviewer for the time and effort spent on reviewing our paper. We believe that these comments help us reduce the possible confusion in the text with respect to paper’s novelty and rigor that appear to have arisen due to lack of sufficient clarity in the original version.

| REVIEWER 2 COMMENT | AUTHOR RESPONSE | PAGE NUMBER |
| --- | --- | --- |
| The idea is good and there is a lot of potential in the topic. However, the writing needs improvement in terms of sentence construction, flow and consistency in Abstract, Introduction and Discussion. Also, there are some mistakes which need to be removed. | We thank the reviewer for pointing this out. The text has been revised.  The heading and main sections revised according to the journal format (1. Introduction, 2. Theoretical background 3. Hypotheses, 4. Methodology5. Discussion 6. Conclusion).  We have fixed this mistake in the revised manuscript. | 1-33 |
| Abstract and Introduction  The rationale of the study needs to be refined. It is not clear why the study needs to be done from the strategic HRM professionals’ perspective in the higher education domain. Sentences need to be reworded (e.g line 13 to 16, line 35-38), some vocabulary needs to be modified (e.g. procure in line 20) and consistency in arguments need to be revisited (Line 63-65). | Thank you. The sentences in the abstract and introduction (lines 4,5,12-15, 29-37, 56-60, 62-67) have been rewritten.  We have simplified the arguments and research gap in the revised manuscript. Regarding the research gap:  1. Educational sector was one of the most impacted by COVID, 2. SHRM practitioners are responsible for supporting and helping people for higher commitment. Studies show that EI and PC are important for managers. Therefore, the actuality goes to study SHRM practitioners in HE sector. | 1-3 |
| Theory and Hypothesis  Literature on EI and SHRM is well covered. However, The SHRM variable needs to be conceptualized more clearly, specifically in terms of literature indicating its significance in the higher education domain. Also, OC can be elaborated further in terms of the three components. Arguments for hypotheses need to be more clear and detailed, and with direct reference to higher education sector. | In the revives manuscript, SHRM practices in higher education are elaborated.  OC with three components (affective, continuance and normative) is explained in the revised manuscript.  Higher education sector in the hypotheses is highlighted. | 6,11,13-14 |
| Data Analysis and Discussion  Data analysis is strong Table 2 needs to be labelled appropriately. The table is giving information about Discriminant Validity but doesn’t contain any values representing it. The arguments in discussion need to be more convincing about the results being specific to the context of Covid-19 situation (contribution stated) and contribution to the higher education domain. | Thank you for this comment.  Arguments in the discussion highlighted the revised manuscript (lines: 540-546, 553-555, 564-582, 584-609). It was a general concern of reviewer 3 that were common. Staffing, training, compensation, and performance appraisal in the higher education and post-COVID-19 discussed.  Table 2, has been corrected (line 476). | 26-29  21 |
| General Comments  There seem to be some errors. In some places in analysis, SHRM is mentioned as an independent variable (Section 4.1.2). Also, the paper mentions POS at the end, which is not a variable under study. AOC and OC seem to have been used interchangeably through all the sections of the paper. In some places, the paper is talking about PC of employees, and in other places PC of SHRM practitioners. | Thank you for these comments:  The definition of variables has been revised and highlighted.  POS is deleted from revised manuscript.  Please also kindly note that the OC has been changed to AOC (highlighted).  Because this paper has been focused on the PC of SHRM practitioners | 13,15,33,39 |

| REVIEWER 3 COMMENT | AUTHOR RESPONSE | PAGE NUMBER |
| --- | --- | --- |
| The topic under consideration is intriguing and warrants significant attention from both scholars and practitioners within the realm of higher education, especially following the COVID-19 endemic. The study commences with a compelling introduction that sets a strong foundation. Overall, the research problem addressed in this paper holds substantial practical and academic significance. However, there are some notable issues that require attention. My recommendation for the current version of the paper is to undergo minor revisions. The authors are encouraged to address and provide responses to the following concerns. | Thank you very much.  The manuscript has been corrected point by point according to your recommendations. |  |
| 1. Throughout the manuscript, there are instances where the author has employed subjective language, notably the use of "We." It is advisable for the author to carefully review these instances and consider removing such subjective language to enhance the paper's objectivity | The manuscript has been corrected from language mistakes.  Objective language has been used. The first-person pronoun (I and we) has been eliminated as proposed by referee 4.    (1.Therefore, it could be noted that organizations must not only rely …  2. The world is trying to recover from the COVID-19 pandemic and changes in employment relationships have already been seen.  3. This paper looks at how HRM practitioners can reimagine their own future in terms of managing their employees. Drawing on insights from psychology, the importance of attending to the human side of employment relationships is emphasized). | 1-3 |
| 2. In the theoretical framework, it is essential to establish a more explicit connection to recent research within the higher education sector, particularly in the context of the post-COVID-19 environment. The author has made efforts to engage in discussions across various sectors; however, it is noticeable that the higher education sector is conspicuously absent. | Issues on the post-COVID-19 have been highlighted in the theoretical framework.  Higher education issues have been presented and discussed in the revised manuscript. Most of the issues in SHRM practices have been added to increase their clarity and comprehension. (For example: SHRM in higher education across different countries (lines, 40-60); employees' expectations for different employment classifications (full-time, part-time) in higher education in the post-COVID-19 era; compensation and the challenges posed by the new normality, limited attention has been given to this area in higher education). | 5,7,9,11 |
| 3. The author has repeatedly emphasized the need for SHRM practitioners to address various questions to tackle the challenges outlined. However, there is ambiguity regarding whether all of these questions directly contribute to the outcomes presented in the paper. Furthermore, in the discussion section, these questions are not adequately addressed in the context of the study. It is essential to provide clarity and reframe these questions within the specific context of the research. | SHRM practices including staffing, training, compensation, and performance appraisal have been added to increase their clarity and comprehension.  Questions under conceptualization of SHRM and COVID-19 in the staffing, training, compensation and performance appraisal have been corrected 2.2.1- 2.2.4. for example: Will the post-COVID-19 workplace require different skills and technologies compared to the pre- Covid-19 era? Is removed.  (One question related to different skills and technology deleted from Training and COVID-19. | 6,9 |
| 4. Upon utilizing the provided outer loading, I randomly attempted to compute the Composite Reliability (CR), Average Variance Extracted (AVE), and the square root of AVE. However, it has come to my attention that there is a significant disparity in the calculated values. Here are a few examples for clarity: • For the AOC Construct: CR = 0.780, AVE = 0.47649075, and the square root of AVE = 0.690283094. • For the SHRM_ST Construct: CR = 0.724, AVE = 0.469198, and the square root of AVE = 0.684980292. These calculations indicate a discrepancy that warrants attention and clarification. Please re-analyze and calculate the entire construct to avoid such inconsistency. | All constructs are reanalyzed, and tables have been corrected accordingly (Tables 1,2,5)  The text has been revised.  We have fixed this mistake (AVE, CR) in the revised manuscript (Table 1).  We are extremely grateful to Reviewer 3 for pointing out this problem. We have recalculated the statistics and revised Table 1 and adjusted the text where highlighted. | 21-23  24,25  21 |
| Additionally, it's important to assess reliability, convergent validity and discriminant validity for the overall construct to ensure that they are measuring distinct aspects of the construct and are related to other constructs in the expected ways. | Done,  Table 1: Reliability and convergent validity; Table 2: Discriminant validity (Fornell – Larcker criterion(; Table 3: Discriminant validity of overall constructs are highlighted. | 21,22 |

| REVIEWER 4 COMMENT | AUTHOR RESPONSE | PAGE NUMBER |
| --- | --- | --- |
| 1. Introduction: main points are mentioned, but there are some discrepancies.  (1) Research objective has some ambiguity. research objective should not be confused with implications. research objective is more about the relationship between the two and the many, rather than about implications. It can be changed to "Being a strong sense of commitment among employees can positively promote the organisation's future success. "  (2) Unclear definition of research gap. There is no talk of research gaps in which niche.  (3) Try to avoid the use of first person. | 1. Sentences in the introduction have been corrected ( lines: 29-36).  2. The sentences in two paragraphs in the introduction have been rewritten. The research gap and reasons are listed from number 1-4 under section introduction.  3. The first person and subjective language has been removed to enhance the manuscript's objectivity. For research gap: firstly the situation has been presented; then the research gap has been identified and finally it is presented what will be done to fulfil the gap (lines56-60). | 2  2,3 |
| 2. The literature review is not adequate and there are numerous omissions. (1) The Theoretical framework needs further definition of relevant involved concepts or variables, i.e., you need to use the definition of concepts in this paper. For example, "In this study, EI is defined as..." (2) Some definitional errors. For example, "Strategic Human Resource Management (first introduced in the 1990s) " Reference: [1]Hartel, C. E. J., & Fujimoto, Y. (2014). Human Resource Management (3rd ed.). Pearson Education. [2]Roehl, M. T. (2019). The impact of SHRM on the psychological contract of employees: A typology and research agenda. Personnel Review, 48(6), 1580–1595. https://doi.org/10.1108/pr-02-2018-0063 [3]Wright, P. M., & Steinbach, A. L. (2022). Pivoting after almost 50 years of SHRM research: toward a stakeholder view. Asia Pacific Journal of Human Resources, 60(1), 22–40. https://doi.org/10.1111/1744-7941.12308 | 1. Sincere apologies for the errors,  the revised manuscript contains EI definition. (in this study EI is defined and introduced as the ability to accurately…. line 78.  2. Definition of SHRM has been corrected based on Wright, P. M., & Steinbach, A. L. (2022) (line 101). | 4  5 |
| (3) Many parts lack relevant literature review and do not do a good work on literature search. (i) In the section of SHRM, there are many opinions and definitions, but there is a lack of relevant theoretical support. (ii) In part 2.2.3, how to find out the form of "Compensation"? Some literature review and summarisation is needed; | 3. Literature related to theoretical framework added (20 more references listed).  (i) This part has been added in the SHRM  (ii) Types of compensation has been explained (section 2.2.3) | 4,5,11  8 |
| (4) Inappropriate placement of the question. Try to avoid using a lot of questions in section 2.2.3. | As advised by Reviewer 3 and mentioned above, all questions under conceptualization of SHRM and COVID-19 have been corrected 2.2.1- 2.2.4. | 6-9 |
| 3. Overall, there are some research contributions, but not much. A large number of articles have been published to study the relationship between the elements of SHRM, EI, PC and so on, while the research contribution of this article focuses on the specific practice field or industrial, and the contribution to the related theories is not much. Further contributions to relevant theories need to be strengthened if further publications are required. | Two paragraphs under section 6.1 (theoretical implications) have been revised (e.g., the study contributes to the broader application of social exchange theory to psychological contract explaining relationship and interaction between employees and employers). | 30-31 |

| REVIEWER 5 COMMENT | AUTHOR RESPONSE | PAGE NUMBER |
| --- | --- | --- |
| Thank you for submitting your paper to this journal. I read your paper and gave my concern down here:  1. It seems that the title is too long and thus, the reader or audience might loose their focuses while reading. I am recommending to shorten it. | Thank you very much for your comments.  The word count of the title has been reduced by using abbreviation SHRM instead of full words.  (SHRM practitioners’ emotional intelligence and affective organizational commitment in higher education during post-COVID-19) | 1 |
| 2. The abstract is not exhaustive. I will suggest to simplify and follow IMRaD concept to present it. | This point was raised by the second reviewer and has been addressed in the revised manuscript. Introduction, Methods, Results, and Discussion are highlighted. | 1 |
| 3. The research problem is not adequately justified. | The research gap has been explained in the revised manuscript (At the moment there are just few studies published in post-COVID-19 environment in educational sector. However, they more focus on well-being …. This study particular focuses not on educators but on SHRM practitioners of educational sector and explores their EI, PC and AOC. | 2,3 |
| 4. Would you add CMB issue as self-reports are there. | Yes, common method bias has been added to limitation as it was self-reported study (6.3. first paragraph). If a VIF exceeds 3.3, it may indicate collinearity and common method bias in the model (Kock, 2015). All VIF values in the inner model resulting from a collinearity test are below 3.3, indicating that the model is free from common method bias. The model is free from common method bias (attached table). \| VIF; EI -> EI_DM \| 1; EI -> EI_SA \| 1; EI -> EI_SAPB \| 1; EI -> EI_SMM \| 1; EI -> SHRM \| 1; PC -> AOC \| 1.854; PC -> PC_RA \| 1; PC -> PC_TRANS \| 1; SHRM -> AOC \| 1.854; SHRM -> PC \| 1; SHRM -> SHRM_CO \| 1; SHRM -> SHRM_PA \| 1; SHRM -> SHRM_ST \| 1; SHRM -> SHRM_TR \| 1 Kock, N. (2015). Common method bias in PLS-SEM: A full collinearity assessment approach. *International Journal of e-Collaboration (ijec)*, *11*(4), 1-10. | 32 |
